# 5-Amino-4-bromo-2,3-dihydro-1*H*-inden-1-one

**DOI:** 10.1107/S1600536812007489

**Published:** 2012-02-24

**Authors:** Ísmail Çelik, Mehmet Akkurt, Makbule Yılmaz, Ahmet Tutar, Ramazan Erenler, Santiago García-Granda

**Affiliations:** a Department of Physics, Faculty of Arts and Sciences, Cumhuriyet University, 58140 Sivas, Turkey; b Department of Physics, Faculty of Sciences, Erciyes University, 38039 Kayseri, Turkey; c Department of Chemistry, Faculty of Art and Science, Sakarya University, 54187 Adapazarı, Turkey; d Department of Chemistry, Faculty of Art and Science, Gaziosmanpaşa University, 60240 Tokat, Turkey; e Departamento Química Física y Analítica, Facultad de Química, Universidad Oviedo, C/ Julián Clavería, 8, 33006 Oviedo (Asturias), Spain

## Abstract

In the title compound, C_9_H_8_BrNO, the non-H-atom framework is essentially planar, with a maximum deviation of 0.087 (3) Å. In the crystal, mol­ecules are inter­connected into a three-dimensional network by C—H⋯O and N—H⋯O hydrogen bonds. In addition, C—H⋯π inter­actions and a π–π stacking inter­action, with a centroid–centroid distance of 3.5535 (19) Å, are also observed.

## Related literature

For bond-length data, see: Allen *et al.* (1987 ▶).
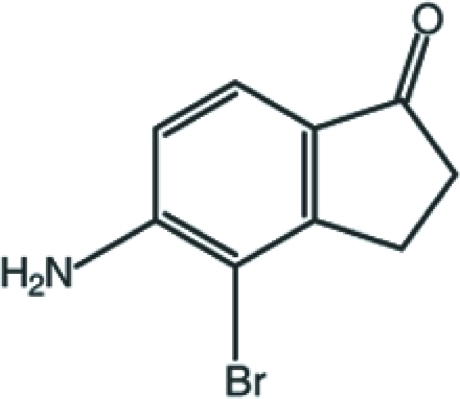


## Experimental

### 

#### Crystal data

C_9_H_8_BrNO
*M* *_r_* = 226.06
Monoclinic, 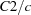

*a* = 12.6362 (4) Å
*b* = 8.3655 (2) Å
*c* = 17.4913 (5) Å
β = 113.128 (4)°
*V* = 1700.37 (10) Å^3^
*Z* = 8
Cu *K*α radiation
μ = 6.16 mm^−1^
*T* = 297 K
0.77 × 0.60 × 0.08 mm

#### Data collection

Agilent Xcalibur Ruby Gemini diffractometer
Absorption correction: multi-scan (*CrysAlis PRO*; Agilent, 2011 ▶) *T*_min_ = 0.031, *T*_max_ = 0.616
3085 measured reflections
1594 independent reflections
1544 reflections with *I* > 2σ(*I*)
*R*_int_ = 0.026

#### Refinement

*R*[*F* ^2^ > 2σ(*F* ^2^)] = 0.049
*wR*(*F* ^2^) = 0.134
*S* = 1.06
1594 reflections
117 parameters
3 restraints
H atoms treated by a mixture of independent and constrained refinement
Δρ_max_ = 1.31 e Å^−3^
Δρ_min_ = −0.89 e Å^−3^

### 

Data collection: *CrysAlis PRO* (Agilent, 2011 ▶); cell refinement: *CrysAlis PRO*; data reduction: *CrysAlis PRO*; program(s) used to solve structure: *SHELXS97* (Sheldrick, 2008 ▶); program(s) used to refine structure: *SHELXL97* (Sheldrick, 2008 ▶); molecular graphics: *ORTEP-3 for Windows* (Farrugia, 1999 ▶); software used to prepare material for publication: *WinGX* (Farrugia, 1997 ▶) and *PLATON* (Spek, 2009 ▶).

## Supplementary Material

Crystal structure: contains datablock(s) global, I. DOI: 10.1107/S1600536812007489/fj2519sup1.cif

Structure factors: contains datablock(s) I. DOI: 10.1107/S1600536812007489/fj2519Isup2.hkl

Supplementary material file. DOI: 10.1107/S1600536812007489/fj2519Isup3.cml

Additional supplementary materials:  crystallographic information; 3D view; checkCIF report

## Figures and Tables

**Table 1:** Hydrogen-bond geometry (Å, °) *Cg*2 is the centroid of the C1–C6 benzene ring.

*D*—H⋯*A*	*D*—H	H⋯*A*	*D*⋯*A*	*D*—H⋯*A*
N1—H1*N*⋯O1^i^	0.83 (4)	2.14 (5)	2.915 (4)	155 (4)
C8—H8*B*⋯O1^ii^	0.97	2.50	3.448 (4)	166
C7—H7*B*⋯*Cg*2^iii^	0.97	2.85	3.659 (4)	141

Symmetry codes: (i) 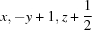; (ii) 
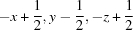
; (iii) 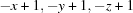.

**Fig. 1.**
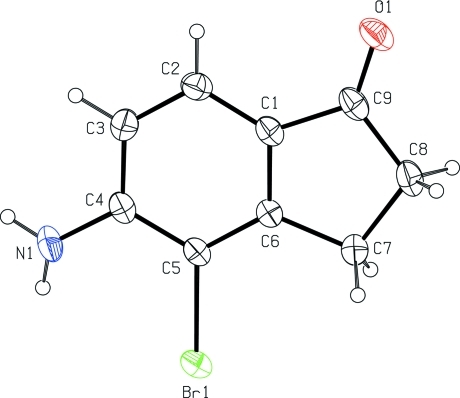
The molecular structure of (I) with the atom numbering scheme. Displacement ellipsoids for non-H atoms are drawn at the 30% probability level.

**Fig. 2.**
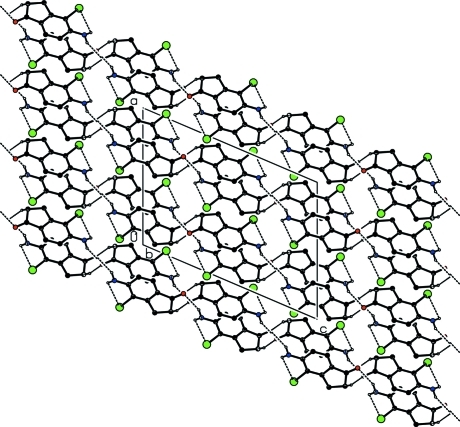
The packing and hydrogen bonding of (I), viewing down the b axis. H atoms not involved in hydrogen bonding have been omitted.

## supplementary crystallographic information

### Comment

In ^1^H-NMR spectrum, H7 appeared at δ 7.53 as a doublet with coupling constant 8.3 Hz and H6 appeared at δ 6.73 (*J* = 8.3 Hz) as doublet. NH2 protons were observed at δ 4.82 with broad singlet. Two signal groups (δ 2.98, δ 2.70) observed at aliphatic region fit with the aliphatic protons. In the present study, we describe the molecular and crystal structures of 5-amino-4-bromo-2,3-dihydro-1*H*-inden-1-one (I), using X-ray diffraction.

As shown in Fig. 1, the molecule of (I), except H atoms, is essentially planar with a maximum deviation of -0.087 (3) Å for O1 atom. Bond lengths and angles observed in (I) are normal (Allen *et al.*, 1987).

The crystal packing is stabilized by intermolecular C—H···O and N—H···O hydrogen bonds (Table 1, Fig. 2) forming a three-dimensional network. In addition, π-π stacking interactions [centroid-centroid distance = 3.5535 (19) Å] between the centroids of the C1–C6 benzene rings of the neighbouring molecules stacking interactions are also observed. C—H···π interactions further help in stabilizing the supramolecular structure (Table 1).

### Experimental

To a stirred solution of 5- acetoaminoindanone (0.4 g, 1.95 mmol) in PEG (2.5 g) was added NBS (1.0 g, 5.6 mmol), SiO_2_ (1.0 g) and NaClO_4_ (0.2 g). The reaction mixture was stirred for 30 days at room temperature, diluted with water (15 ml), extracted with diethyl ether (3×25 ml), dried (Na_2_SO_4_). After removal of the solvent, the residue was chromatographed on silica gel eluted with chloroform/hexane (4/1) afforded the title compound which was crystallized from dichloromethane-hexane yielded the colourless plate crystal (0.11 g, 25%), ^1^H-NMR (400 MHz, CDCl_3_); δ 7.53 (d, *J* = 8.3 Hz, 1H, H7), 6.74 (d, *J* = 8.3 Hz, 1H, H6), 4.82 (brs, 2H, NH_2_), 2.98 (m, 2H, H2), 2.70 (m, 2H, H3).

### Refinement

The H atoms of the amino group was located in a difference Fourier map and were isotropically refined with the distance restraints (N—H = 0.86 (2) Å and H···H = 1.30 (2) Å). C-bound H-atoms were positioned geometrically and refined using a riding model [C—H = 0.93 and 0.97 Å, and *U*_iso_(H) = 1.2*U*_eq_(C)]. The highest residual electron density peak and the deepest hole are located 1.03 Å and 0.89 Å from Br1, respectively.

### Crystal data

**Table:**

C_9_H_8_BrNO	*F*(000) = 896
*M**_r_* = 226.06	*D*_x_ = 1.766 Mg m^−^^3^
Monoclinic, *C*2/*c*	Cu *K*α radiation, λ = 1.5418 Å
Hall symbol: -C 2yc	Cell parameters from 2518 reflections
*a* = 12.6362 (4) Å	θ = 5.5–70.2°
*b* = 8.3655 (2) Å	µ = 6.16 mm^−^^1^
*c* = 17.4913 (5) Å	*T* = 297 K
β = 113.128 (4)°	Plate, colourless
*V* = 1700.37 (10) Å^3^	0.77 × 0.60 × 0.08 mm
*Z* = 8	

### Data collection

**Table:**

Agilent Xcalibur Ruby Gemini diffractometer	1594 independent reflections
Radiation source: Enhance (Cu) X-ray Source	1544 reflections with *I* > 2σ(*I*)
Graphite monochromator	*R*_int_ = 0.026
Detector resolution: 10.2673 pixels mm^-1^	θ_max_ = 70.4°, θ_min_ = 5.5°
ω scans	*h* = −13→15
Absorption correction: multi-scan (*CrysAlis PRO*; Agilent, 2011)	*k* = −6→10
*T*_min_ = 0.031, *T*_max_ = 0.616	*l* = −19→21
3085 measured reflections	

### Refinement

**Table:**

Refinement on *F*^2^	Primary atom site location: structure-invariant direct methods
Least-squares matrix: full	Secondary atom site location: difference Fourier map
*R*[*F*^2^ > 2σ(*F*^2^)] = 0.049	Hydrogen site location: inferred from neighbouring sites
*wR*(*F*^2^) = 0.134	H atoms treated by a mixture of independent and constrained refinement
*S* = 1.06	*w* = 1/[σ^2^(*F*_o_^2^) + (0.1063*P*)^2^ + 1.0273*P*] where *P* = (*F*_o_^2^ + 2*F*_c_^2^)/3
1594 reflections	(Δ/σ)_max_ = 0.001
117 parameters	Δρ_max_ = 1.31 e Å^−^^3^
3 restraints	Δρ_min_ = −0.89 e Å^−^^3^

### Special details

**Table:**

Geometry. Bond distances, angles *etc*. have been calculated using the rounded fractional coordinates. All su's are estimated from the variances of the (full) variance-covariance matrix. The cell e.s.d.'s are taken into account in the estimation of distances, angles and torsion angles
Refinement. Refinement on *F*^2^ for ALL reflections except those flagged by the user for potential systematic errors. Weighted *R*-factors *wR* and all goodnesses of fit *S* are based on *F*^2^, conventional *R*-factors *R* are based on *F*, with *F* set to zero for negative *F*^2^. The observed criterion of *F*^2^ > σ(*F*^2^) is used only for calculating -*R*-factor-obs *etc*. and is not relevant to the choice of reflections for refinement. *R*-factors based on *F*^2^ are statistically about twice as large as those based on *F*, and *R*-factors based on ALL data will be even larger.

### Fractional atomic coordinates and isotropic or equivalent isotropic displacement parameters (Å^2^)

**Table:**

	*x*	*y*	*z*	*U*_iso_*/*U*_eq_	
Br1	0.52857 (3)	0.21900 (4)	0.63347 (2)	0.0470 (2)	
O1	0.2303 (2)	0.4628 (3)	0.26770 (13)	0.0544 (8)	
N1	0.3501 (3)	0.4337 (4)	0.66390 (17)	0.0535 (9)	
C1	0.3037 (3)	0.4330 (4)	0.41603 (17)	0.0381 (8)	
C2	0.2318 (3)	0.5262 (4)	0.44160 (18)	0.0422 (8)	
C3	0.2489 (3)	0.5253 (4)	0.5240 (2)	0.0431 (9)	
C4	0.3357 (3)	0.4322 (4)	0.58293 (17)	0.0399 (8)	
C5	0.4086 (2)	0.3422 (3)	0.55556 (16)	0.0368 (8)	
C6	0.3917 (2)	0.3426 (3)	0.47291 (16)	0.0345 (8)	
C7	0.4597 (3)	0.2542 (4)	0.4321 (2)	0.0430 (9)	
C8	0.4016 (3)	0.3016 (4)	0.3399 (2)	0.0493 (10)	
C9	0.3006 (3)	0.4086 (4)	0.33249 (17)	0.0425 (8)	
H1N	0.301 (3)	0.472 (5)	0.679 (3)	0.062 (12)*	
H2	0.17350	0.58760	0.40350	0.0510*	
H2N	0.387 (4)	0.369 (6)	0.701 (3)	0.10 (2)*	
H3	0.20180	0.58790	0.54150	0.0520*	
H7A	0.45550	0.13960	0.43890	0.0520*	
H7B	0.53980	0.28670	0.45540	0.0520*	
H8A	0.45530	0.35870	0.32260	0.0590*	
H8B	0.37490	0.20740	0.30530	0.0590*	

### Atomic displacement parameters (Å^2^)

**Table:**

	*U*^11^	*U*^22^	*U*^33^	*U*^12^	*U*^13^	*U*^23^
Br1	0.0466 (3)	0.0552 (4)	0.0315 (3)	0.0060 (1)	0.0070 (2)	0.0028 (1)
O1	0.0541 (13)	0.0741 (16)	0.0303 (10)	−0.0051 (12)	0.0114 (10)	0.0072 (10)
N1	0.0619 (17)	0.0712 (18)	0.0322 (13)	0.0012 (15)	0.0237 (13)	−0.0070 (12)
C1	0.0412 (14)	0.0433 (13)	0.0290 (13)	−0.0056 (12)	0.0128 (11)	0.0001 (10)
C2	0.0393 (14)	0.0471 (15)	0.0368 (15)	0.0014 (12)	0.0114 (12)	0.0050 (12)
C3	0.0443 (15)	0.0451 (14)	0.0443 (16)	0.0013 (13)	0.0221 (13)	−0.0033 (12)
C4	0.0444 (14)	0.0451 (14)	0.0305 (14)	−0.0062 (12)	0.0152 (12)	−0.0046 (11)
C5	0.0386 (13)	0.0414 (14)	0.0274 (12)	−0.0026 (12)	0.0098 (10)	−0.0017 (10)
C6	0.0371 (13)	0.0362 (13)	0.0298 (13)	−0.0009 (10)	0.0126 (11)	−0.0008 (10)
C7	0.0438 (17)	0.0496 (13)	0.0376 (17)	0.0024 (14)	0.0182 (14)	−0.0045 (13)
C8	0.0578 (19)	0.0602 (18)	0.0344 (16)	−0.0064 (15)	0.0230 (15)	−0.0064 (13)
C9	0.0474 (15)	0.0501 (15)	0.0299 (14)	−0.0140 (13)	0.0151 (12)	−0.0006 (11)

### Geometric parameters (Å, º)

**Table:**

Br1—C5	1.896 (3)	C5—C6	1.376 (4)
O1—C9	1.220 (4)	C6—C7	1.509 (5)
N1—C4	1.355 (4)	C7—C8	1.539 (5)
N1—H1N	0.83 (4)	C8—C9	1.522 (5)
N1—H2N	0.83 (5)	C2—H2	0.9300
C1—C9	1.461 (4)	C3—H3	0.9300
C1—C2	1.397 (5)	C7—H7A	0.9700
C1—C6	1.389 (4)	C7—H7B	0.9700
C2—C3	1.371 (4)	C8—H8A	0.9700
C3—C4	1.409 (5)	C8—H8B	0.9700
C4—C5	1.411 (5)		
			
H1N—N1—H2N	105 (5)	C7—C8—C9	106.3 (3)
C4—N1—H1N	122 (3)	O1—C9—C1	126.9 (3)
C4—N1—H2N	128 (3)	O1—C9—C8	125.3 (3)
C2—C1—C6	120.9 (3)	C1—C9—C8	107.8 (3)
C2—C1—C9	129.3 (3)	C1—C2—H2	121.00
C6—C1—C9	109.9 (3)	C3—C2—H2	121.00
C1—C2—C3	118.7 (3)	C2—C3—H3	119.00
C2—C3—C4	121.9 (3)	C4—C3—H3	119.00
C3—C4—C5	118.1 (3)	C6—C7—H7A	111.00
N1—C4—C5	121.5 (3)	C6—C7—H7B	111.00
N1—C4—C3	120.4 (3)	C8—C7—H7A	111.00
Br1—C5—C4	119.4 (2)	C8—C7—H7B	111.00
Br1—C5—C6	120.5 (2)	H7A—C7—H7B	109.00
C4—C5—C6	120.2 (2)	C7—C8—H8A	110.00
C1—C6—C5	120.3 (3)	C7—C8—H8B	111.00
C1—C6—C7	111.9 (3)	C9—C8—H8A	110.00
C5—C6—C7	127.9 (3)	C9—C8—H8B	110.00
C6—C7—C8	104.1 (3)	H8A—C8—H8B	109.00
			
C6—C1—C2—C3	−0.8 (5)	N1—C4—C5—Br1	0.6 (4)
C9—C1—C2—C3	177.4 (4)	N1—C4—C5—C6	179.8 (3)
C2—C1—C6—C5	0.7 (5)	C3—C4—C5—Br1	178.7 (2)
C2—C1—C6—C7	−178.9 (3)	C3—C4—C5—C6	−2.1 (5)
C9—C1—C6—C5	−177.8 (3)	Br1—C5—C6—C1	180.0 (2)
C9—C1—C6—C7	2.7 (4)	Br1—C5—C6—C7	−0.5 (4)
C2—C1—C9—O1	−2.6 (6)	C4—C5—C6—C1	0.8 (4)
C2—C1—C9—C8	177.6 (4)	C4—C5—C6—C7	−179.7 (3)
C6—C1—C9—O1	175.7 (3)	C1—C6—C7—C8	−0.1 (4)
C6—C1—C9—C8	−4.1 (4)	C5—C6—C7—C8	−179.7 (3)
C1—C2—C3—C4	−0.7 (5)	C6—C7—C8—C9	−2.4 (3)
C2—C3—C4—N1	−179.8 (4)	C7—C8—C9—O1	−175.9 (3)
C2—C3—C4—C5	2.1 (5)	C7—C8—C9—C1	4.0 (4)

### Hydrogen-bond geometry (Å, º)

Cg2 is a centroid of the C1–C6 benzene ring.

**Table:**

*D*—H···*A*	*D*—H	H···*A*	*D*···*A*	*D*—H···*A*
N1—H1*N*···O1^i^	0.83 (4)	2.14 (5)	2.915 (4)	155 (4)
N1—H2*N*···Br1	0.83 (5)	2.80 (5)	3.088 (4)	103 (4)
C8—H8*B*···O1^ii^	0.97	2.50	3.448 (4)	166
C7—H7*B*···*Cg*2^iii^	0.97	2.85	3.659 (4)	141

Symmetry codes: (i) *x*, −*y*+1, *z*+1/2; (ii) −*x*+1/2, *y*−1/2, −*z*+1/2; (iii) −*x*+1, −*y*+1, −*z*+1.
